# Something looks fishy! A philosophical exploration of AI for marine conservation

**DOI:** 10.1007/s43681-026-01312-y

**Published:** 2026-08-31

**Authors:** Mark Ryan, Paulan Korenhof

**Affiliations:** Wageningen University and Research, Wageningen, Netherlands

**Keywords:** Society, Values, Animal welfare, Technology, Post-phenomenology

## Abstract

Increased pollution, traffic, overfishing, and climate change threaten fish populations, their habitats, and the sustainability of marine ecosystems. To develop environmental policies that support the conservation of sustainable ocean ecosystems and foster biodiversity, the use of artificial intelligence (AI) is proposed to improve knowledge production on various fish species. These fishial recognition systems (FRS) integrate machine learning, deep learning, convolutional neural networks, computer vision, drones, and robots to identify fish species, monitor endangered populations, track migration patterns, detect illegal fishing, and even track and kill invasive species. While these FRS are employed for biodiversity protection, they may change practices, shift relations, and shape the production of new knowledge and access thereto. The technical community actively adopts FRS, but the ways this technology affects fish, humans, and their shared environments remain underexplored. Therefore, this article conducts philosophically informed, exploratory research into the implications of FRS to address the central research question: how do FRS affect fish in open waters? This paper analyses the technical literature and draws on various bodies of philosophical and social science research to identify the main social and ethical considerations of FRS. It identifies potential (intentional and unintentional) biases found in the data, algorithms, or applications of FRS. It also explores the potential physical and emotional harm to fish caused by underwater FRS. FRS may further exacerbate anthropocentric, quantification, and power asymmetries between humans and fish. In response, this paper develops a fish-centric approach to the development, deployment, and use of FRS.

## Introduction

Increased pollution, traffic, overfishing, and climate change threaten marine biodiversity [17, 76]. Currently, multiple fish species worldwide are threatened with extinction [60]. Nevertheless, diversity in fish species is vital for a sustainable ocean ecology [9, 35, 64]. To develop environmental policies that support sustainable ocean ecosystems and foster biodiversity, many are exploring ways to improve knowledge production about different fish species [1, 31]. As oceans are a complex environment for human data collection, some have turned to the development, implementation, and use of conservation technologies, particularly AI-assisted tools, to achieve this [48, 65, 86, 91]. This paper will focus on one of these conservation technologies: AI-based fish recognition.

Fishial recognition systems (hereafter, FRS) are AI-driven computer vision systems that identify fish through image-based pattern recognition. Like facial recognition systems used on humans, FRS commonly employ convolutional neural networks (CNNs) to achieve visual identification and categorisation. However, these deep learning networks are trained on large datasets of fish images to identify and categorise species based on visual markers such as body patterns, body shape and scale configurations (rather than faces, ala facial recognition). Applications of FRS include species identification, monitoring endangered populations, tracking migration patterns, detecting illegal fishing, and even tracking and killing invasive species [87, 91]. Additionally, in the challenging marine environment, these systems significantly increase the speed, extent and timeliness of biodiversity monitoring [14, 30].

While these FRS are employed for biological conservation, they are, like all technologies, not neutral: as technologies are produced, implemented and used, they change practices, shift relations, and shape the production of new knowledge and the access thereto [32, 51]. FRS are likely to affect those who come into contact with them during their design, production, implementation, or use, including the fish themselves. While FRS is actively adopted by the technical community, the ways this technology affects fish, humans, and their shared environments remain underexplored. Therefore, this article conducts philosophically informed, exploratory research into the implications of FRS to address the central research question: how do FRS affect fish in open waters?

To explore the implications of FRS, we first analyse the technical literature to identify the main characteristics of these systems, with a focus on FRS in open waters. While FRS is being implemented in aquaculture, this paper focuses specifically on its use as a preventive tool for biodiversity monitoring and conservation. While the use of FRS in aquaculture raises many ethical issues (e.g., the ethics of breeding, confining, and controlling fish solely for consumption), it is beyond the scope of this paper.

The second section of this paper outlines the methodology used to retrieve literature on FRS, while Sect. 3 focuses on the analysis of the articles. Section 4 identifies three overarching philosophical considerations about the impact of FRS in open waters: informational, infrastructural, and relational dimensions. Section 5 focuses on considerations and potential responses to address these three-dimensional concerns. Lastly, the paper concludes by highlighting the main lessons learned and offers an agenda for a fish-centric approach to FRS and further research needed in this area.

## Methodology

The first step in this paper is conducting a literature review of articles on the use of computer vision technologies for fish identification and categorisation in open waters. The purpose of this literature search was to identify the technologies deployed in the field, the state-of-the-art research being conducted, the fish identified and categorised, and the bodies of water in which the research is conducted. This information provides a backdrop for analysing the philosophical implications of FRS. Therefore, a literature search in Scopus was conducted using the following search query:

(TITLE-ABS-KEY(fish) AND TITLE-ABS-KEY(“computer vision”) OR TITLE-ABS-KEY(“object detection”) OR TITLE-ABS-KEY(“object recognition”) OR TITLE-ABS-KEY(“video tracking”) OR TITLE-ABS-KEY(“activity recognition”) AND TITLE-ABS-KEY(ocean) OR TITLE-ABS-KEY(marine) OR TITLE-ABS-KEY(river) OR TITLE-ABS-KEY(sea) AND NOT TITLE-ABS-KEY(farm) AND NOT TITLE-ABS-KEY(aquaculture) AND NOT TITLE-ABS-KEY(breeding)) AND PUBYEAR > 2022 AND PUBYEAR < 2026 AND ( LIMIT-TO ( SRCTYPE, “j”) OR LIMIT-TO ( SRCTYPE, “p”)) AND ( LIMIT-TO ( PUBSTAGE, “final”)) AND ( LIMIT-TO ( LANGUAGE, “English”)).

The first keyword in this search query was used to ensure that the articles were specifically focusing on fish. The second keyword group was used to find papers that specifically deploy computer vision technologies on fish. As the focus of this paper is on FRS, keywords related to computer vision and object detection were used here. The third group of keywords was used to narrow our search to articles focusing on open waters, rather than fish farms or aquaculture (which we also included as restrictions in the search query).

Additionally, the search query was limited to articles published after 2022 to ensure only the most current, state-of-the-art research was included. Because the field changes so fast, it was best to capture what the current research says on the topic. In addition, the purpose of the literature review is to provide a snapshot and background of current research, rather than to function as a systematic review or an exhaustive mapping of the field.

This search query brought back 121 articles. Four exclusion criteria were applied to further refine this list. Articles were excluded if they did not focus on alive fish (some articles focused on fish weight at markets, etc.) (40), did not focus on computer vision (some articles used older approaches such as sonar) (16), did not focus on open waters (some articles focused on aquariums or fish farms) (7), or were review articles (3). This process resulted in 55 articles for retrieval. However, due to licensing and accessibility issues, 25 articles were unretrievable,^1^ leaving 30 articles for review (see Appendix 1). While the number of articles available for analysis was lower than desirable, there was considerable convergence among the articles, with most being descriptive scientific papers on the technical methods for using FRS. This literature review aimed to provide general insights into how these technologies are implemented in practice and to identify the philosophical underpinnings of their application. Although all 30 eligible studies were analysed, the synthesis reached a point of diminishing returns after approximately 10–15 studies, with subsequent papers largely confirming previously identified findings rather than contributing new themes.

The 30 articles were thematically analysed to identify the types and purposes of technologies being deployed, the types of fish being analysed, the datasets used, the location of the analysis, the reasons underpinning the research, and potential challenges associated with FRS [13, 85]. The aim of this is to provide a clear overview of FRS research to assess its philosophical implications. The review is not meant as a comprehensive mapping of the entire field, nor to provide an evaluation of the technical aspects of these computer science papers. Instead, to give an overview of these technologies to lend insight into potential philosophical analysis.

For the philosophical analysis of FRS, we draw on three bodies of literature that reflect three angles on analysing the technology: the informational, the infrastructural, and the relational.

First, FRS is an *information* technology with the primary aim of producing information. We will therefore first explore the implications arising from and in the *informational dimension*, tying in to literature from critical data studies and AI ethics [26, 44]*.* The *informational dimension* starts with the fact that a large part of the current challenges in systems revolves around producing and accessing sufficient, high-quality data [25]. Before using this data for computer vision applications, it must be processed, cleaned, and uploaded to the system. However, this process is often situated in a particular region and with a specific ecological, geographical, and cultural context.

Second, as technologies, FRS are socio-material constructs that tie into many processes and other technologies to function. The technology thus relies on an infrastructure embedded in particular equipment, logistics, actors, and practices that support, in our case, the production of knowledge [12]. For example, FRS relies on large-scale infrastructure to produce and process relevant data. This dimension is driven by the prevalence of hardware devices (e.g., underwater cameras, sonar systems, underwater drones, and robots) used for data collection. Therefore, it is best to approach and describe such technologies as a heterogeneous assembly of diverse components that together form a larger whole (i.e., FRS) [73]. Therefore, the second dimension we will discuss is the *infrastructural dimension*. Here, we draw on the literature from infrastructure studies and assemblage theory (see, e.g., [12, 73]).

Thirdly, FRS ties into the environment of both fish and humans, and establishes and shapes relations between humans and fish. The third dimension we will therefore explore is the *relational* dimension. The relation established by FRS could have a significant impact on the human-animal relationship [18]. This ties in to a body of literature on/from animal and environmental ethics [10, 11].

Section 2 highlights this analysis and provides a detailed background of FRS.

## Fishial recognition systems

The identification, counting, and monitoring of fish have undergone many different stages over the years. Initially, it involved ‘traditional counting methods’, which primarily relied on fishers estimating the number of fish by catching them [46]. However, this proved to be very time-consuming and labour-intensive, ‘while also impacting the welfare and growth of the fish’ [46], p. 2). Another approach was the use of sensors, infrared optical counters, and resistivity fish counters to identify and count fish species [46], p. 2). Optical and acoustic methods were deployed but were unsuccessful for classifying and categorising fish [46], p. 2). As a result of the deficiencies of these two approaches, there has been an influx of research taking place on the use of ‘deep learning-based underwater video automatic resource counting methods for identification and enumeration’ [46], p. 2).

The use of artificial intelligence (AI) methods and rapid advancements in hardware technologies have enabled researchers to develop approaches to fish identification, counting, and categorisation. Hardware devices such as underwater cameras, underwater drones, and robots have enabled the collection of vast amounts of images and data on fish activity in open waters. They also enable real-time visualisation of underwater activities, enabling immediate action and responses to promote biodiversity conservation and restoration. With the rise of Digital Twin development for marine management, like Europe’s Digital Twin of the Ocean project,^2^ we expect that the use of fishial recognition systems in the future will often happen in tandem with projects that aim to support fishery management, sand extraction, or wind energy. As such projects can pose risks to marine biodiversity, the collection of real-time marine biodiversity data is expected to reduce the harm these projects cause to biodiversity and better align them with the United Nations’ sustainable development goals. While not yet regularly employed, we expect to see a rise in fishial recognition systems for biodiversity and conservation purposes in the future.

Computer vision, a category of AI, has been deployed to detect, locate, and classify objects, and to provide insights into the phenomena under examination. FRS is often based on two categories of computer vision technology: traditional hand-designed approaches and deep learning approaches [46]. However, most traditional computer vision methods use hand-designed operators to extract fish features, and different operators need to be designed for different categories of fish, which generally has the disadvantages of complex detection and poor generalisation. In addition to this, in the recognition process, it is also susceptible to the interference of environmental factors, such as light, background, etc., and there are problems such as difficulty in feature extraction and poor robustness [46], p. 2).

Therefore, deep learning-based object detection has become increasingly significant for fish analysis in recent years. Deep learning object detection defines bounding boxes around objects and assigns each a category. These bounding boxes can be quantified and categorised to produce valuable information about the number and types of fish. Several architectures and approaches are being implemented for this process, most commonly CNNs. A CNN extracts high-level features, while a classification network identifies fish species, clusters them, and counts them based on these features. These use ‘many hidden layers with neuron pooling constraints and regularisation with maximum value are used to detect the objects from the frames’ [8], p. 87,120). Some examples of CNNs used in the literature include PFFS-Net [34], UNet [16, 66], DeepLabV3 + [16], GhostNet [82], AlexNet [20], RetinaNet [14], and ResNet-50 [20].

Furthermore, CNN-based object detection is often divided into two approaches: one- or two-stage models. An example of a one-stage model is the You Only Look Once (YOLO) algorithm, which ‘is an end-to-end model, directly regressing and outputting the positions and categories of detected objects’ [46], p. 3).^3^ From the literature analysed, researchers used YOLOv4 [3], YOLOv5 [47, 81, 83],J. [89], YOLOv5s [82], YOLOv7 [3, 48], YOLOv8 [5, 24, 49, 84],J. [89], YOLOv8m [4], YOLOv10 [14], YOLOv11 [40].

Two-step models, such as Fast R-CNN [14], Faster R-CNN [24] and Mask R-CNN, use a ‘two-step strategy involving anchor box filtering from coarse to fine and regression from fine to coarse. This strategy sacrifices speed to enhance precision’ [46], p. 3). For example, the Foreground Region Convolution Network (FR-CNN) is a two-stage object detection method that combines supervised and unsupervised learning techniques [46]. This method begins with image pre-processing and enhancement techniques to improve image clarity. The model then implements foreground segmentation to ‘isolate potential fish targets from the background, and background subtraction techniques are used to identify regions of motion and serve as candidate regions for further analysis by the convolutional network’ [46], p. 5). Other researchers combined their CNNs with video tracking algorithms [92], Self-Guided Residual Attention Networks (SGRAN) [7, 8], and a novel hybrid capuchin-based coevolving particle swarm optimisation (HC2PSO) algorithm [50].

In addition to these deep learning approaches, data was used from an array of sources such as open-source datasets [4, 46], video data from a submarine cable online observation system [46], and fish species images from the internet (Kaggle and Roboflow) [5]. At the same time, some researchers collected datasets themselves from GoPro cameras, stationary underwater cameras, and sonar equipment [3, 47, 83, 92], most used accessible fish datasets (see Table 1).

**Table 1:** Most commonly used fish data sets

Data sets used	
Fish4Knowledge [40, 58, 78]	
Fish-Pak [20, 78]	
QUT FISH [7, 8, 78, 88]	
DeepFish [7, 8, 16, 34],J. [89],P. [90]	
Submarine Imagery (SUIM) [16, 34]	
SEAMAPD21 [55, 78]	
WildFish [78, 88]	

Most of the datasets and analyses in the research focused on fish species as a whole rather than a specific region or context. However, several papers concentrated on specific regions or contexts of fish identification, such as the marine environments [58, 82], Dongji Island, Zhejiang Province, China [46, 49], Australia [40],P. [90], Red Sea Coral Reef [47], Eastern parts of India [20], New Caledonia, South Pacific [92], Gulf of Mexico [55], Newfoundland, Canada [3], Peru [66] and Indonesia [5].

Similarly, the types of fish analysed were often general, such as Amazonian fish [66], Mediterranean fish [14], tropical and subtropical fish [49, 58],marine fish near coral reefs [82], freshwater fish [20], or deepwater snapper fish (Lutjanidae family) [92]. While two papers focused on fish, they also analysed other marine animals [4, 84].

The aims of the papers also differed, from detecting fish and analysing fish species behaviour *in relation to environmental conditions* [38, 46], implementing *real-time* detection of fish in maritime surveillance [24], to identifying the population size and growth of fish as its primary objective [82]. However, all articles converged in their aim to ‘automatically and precisely determine fish species from images’ [58], p. 144) and to automatically detect and classify fish species in marine environments [58, 82]. Essentially, fish identification and categorisation were the primary goals of FRS research [4, 5, 7, 8, 20, 50, 84, 92].

## Philosophical analysis of FRS

A wide array of FRS is being deployed to identify and categorise fish. All of the papers focused on the technical dimensions of the research, their effectiveness, and breakthroughs in computational capabilities. Still, they did not provide insight into whether or not they should be implemented in practice, their ethical and social ramifications, or how best to address them. The following sections will explore three dimensions of FRS that emerged from analysis and reflection on the technical FRS literature: informational, infrastructural, and relational.

### Informational dimension

FRS serve to produce information about fish. Nevertheless, the information these systems produce is not neutral: it is *specific* information (e.g., time, place, and amount) about something (e.g., fish) for someone (e.g., conservationists, biologists, and policymakers). Information is produced through an interplay among human decision-makers, technological affordances, and contextual factors [44]. As such, the production of information will in all cases take place within processes of selection and framing. Every materialised piece of information, whether it is printed on paper or displayed on a screen, will have a certain bias: it will refer to particular aspects of the world while omitting others. This bias is needed because one needs a specific focus, or would drown in information overload. At the same time, selection and framing can result in exclusion, blind spots, and errors in the information produced, which, in turn, can be detrimental if we rely on it for decision-making. This section highlights several biases and how they materialise in FRS.

Firstly, there may be biases in how the research is set up. For instance, to build FRS, designers need to interpret and translate the interaction of fish in open waters with a particular materialised computer-vision-based perspective [43]. They need to decide on the system’s design and infrastructure, the location, the timeframe, and the software and hardware to use. These choices may be shaped by biases in the researchers’ knowledge, cultural background, worldviews, and perceptions of the problem [29, 41]. As these choices shape the FRS, they also guide its potential output. For example, if researchers are interested in safeguarding biodiversity, they will likely distribute sensors in locations that align with the behaviour patterns of endangered fish [40] or keystone species [47]. This behaviour can result in data collection that may overlook impacts on *non-endangered* or *non-keystone* fish. Given the goal of safeguarding biodiversity, prioritising endangered species seems sensible; however, if the population of non-endangered species suddenly plummets, this may fall outside the scope due to the research design and the data collected. This effect may catch researchers off guard and prevent them from implementing action before it reaches this stage. The reason for this is that the overemphasis on endangered species distorts their focus.

Secondly, biases in the data gathered for FRS may lead to inaccurate observations, poor decisions, and subsequent harm to fish species or their habitats [67]. While ‘scientists can gather extensive data on fish behaviour, species diversity, and ecosystem health’ [84], p. 1), it is often difficult to retrieve images from the environment in a sufficient quantity and quality. FRS research ‘still faces the problems of poor quality datasets and insufficiently advanced detection algorithms’ [82], p. 3). It is challenging to continuously monitor fish activity in underwater environments, particularly due to the presence of foreign objects, environmental changes, and reduced visibility [46, 69]. Variants, such as ‘low light conditions, scattering, and absorption resulting from suspended particles and algae in the water’ can affect the quality of identification [46], p. 1). Water is denser than air, so the light that enters the water ‘is refracted, absorbed, and scattered’, so the light is ‘dispersed in various directions, resulting in these light drops’ [4], p. 793,[84]. In addition, Floating particles known as marine snow enhance the absorption and scattering effects. By their wavelengths, colours diminish one by one as we descend deeper into the universe. Since blue has the shortest wavelength of all colours and hence goes the furthest in water, blue colour dominates underwater imagery. Furthermore, there is blurring, low contrast, and low brightness [4], p. 793,see also, [84].

Biases and/or inaccuracies in datasets and/or algorithms may lead to false positives and false negatives, skewing representations of fish species numbers, behaviours, and ocean ecosystem health [69]. Such misrepresentations may have severe consequences if FRS results are used in environmental policy decision-making, potentially leading to the misallocation of resources, the failure to recognise endangered species, and an inability to take appropriate action when and where needed.

Thirdly, bias may be intentional, for example, aiming to protect a species threatened with extinction, one considered to be of greater ecological importance, or one that might be ‘charismatic species’^4^ and have been the target of more data-collection efforts [45, 52, 71]. Historically, biodiversity datasets tend to show a bias toward more commonly reported fish species [77]. Bias may also be unintentional, coming from technical or environmental limitations. Based on shape, size, and behaviour, some fish species are easier to analyse than others, leading to a greater presence and visibility in FRS information production [61, 72]. Systems trained on specific species, geographic regions, or lighting conditions may perform better in identifying particular species over others. As a result, researchers may focus only on fish in these more ‘favourable’ conditions (and neglect others), or on particular fish species and conditions where FRS is effective.

### Infrastructural dimension

FRS that conserve and support biodiversity in open waters rely on infrastructure for data generation and processing, including sensors, cameras, and other hardware and software that enable the system to function [41, 74]. The infrastructural dimension of FRS is the manner in which the system materially takes shape in the world (e.g., in open waters). FRS require deploying various technological devices in the ocean to monitor fish species. As such, FRS are part and parcel of what Wickberg and Lidström call ‘environing media’: ‘The digital ocean is the result of massive, while at the same time limited amounts of sensors in the physical ocean’ [80], p. 4473). In the case of FRS, the key elements of the system are camera placement and the use of connecting materials. To be ‘recognised’ by the system, fish need to be recorded or photographed. The cameras can be mounted on boats, drones, or marine platforms, thereby allowing a foreign, moving or unmoving object to enter the marine environment. Recording fish in open water requires installing surveillance architecture, which can have specific impacts on fish, including altering their behaviour and causing physical harm to them or the environment. The relation between animals and their environment is important to consider from an animal ethics perspective because animal welfare is understood as an animal’s positive experiences of its environment, as well as their ability and capacity to adapt to it [11, 68].

In the context of the infrastructural dimension, Bovenkerk and Meijboom’s discussion of ethics and fish welfare is particularly relevant. Following Fraser [28], Bovenkerk and Meijboom discuss three approaches to fish welfare: function-, feeling-, and nature-based approaches [11]. Bovenkerk and Meijboom state: ‘Applied to fish, function-based views are about the ability of fish to cope with farming conditions. Feeling-based views assume that fish have subjective feelings and that these are constitutive of their welfare (…). Nature-based views regard fish welfare as the ability of fish to display natural or species-specific behaviour’ (2020, p. 25). Despite their focus on fish farming, we consider the points they identify helpful and relevant for drawing out the potential implications for FRS infrastructures for oceanic fish.

First, the infrastructure may pose functional challenges to oceanic fish. With ‘functional’ challenges to animal welfare, Fraser refers to the ‘biological functioning of the animal in the sense of health, growth, and productivity’ [28], p. 435). In this context, we consider that the deployment of vessels, robotic fish, drones, and moving cameras may potentially cause physical harm [2, 67, 68]. It has been shown that boats and fishing equipment can strike and harm fish, leak pollutants into the ocean, create noise pollution that physically harms fish, or generate waves that harm fish habitats [15, 79]. There is also the possibility that researchers will need to deploy *more* vessels, robots, and cameras to retrieve images from the ocean to improve FRS. Researchers may begin deploying more technologies in the oceans, potentially leading to an ecosystem saturated with devices collecting images for fish monitoring and protection.

Secondly, FRS may act as stressors to fish. In humans, knowing one is being watched or recorded may lead to the internalisation of specific behaviour [27], a phenomenon that may also occur in fish. However, it is unclear what impact this constant monitoring will have on fish welfare. Fish have varying levels of vision, but most appear to have vision similar to humans’, albeit with the ability to see more colours (e.g., ultraviolet light). However, due to evolution and the need to focus on potential predators, they tend to focus more on *movement* rather than specific objects [33]. However, some species of fish, such as sharks, can see up to 50 feet ahead of them at any given time, ten times farther than humans can underwater [19].

Fish have semicircular canals in their inner ears, which are small tubes filled with fluid: ‘These canals are lined with sensory neurons that float in this fluid and activate whenever they detect movement’ [6], p. 148). When fish move in a certain direction, the fluid in these canals moves. Therefore, this vestibular sense allows fish to position themselves, move, and construct spatial maps in their environment. They use this to distinguish between swimming toward an animal and the animal swimming toward them. The visual cue remains the same, but the vestibular system is activated when the animal swims toward it, not when it swims away from it [6]. Therefore, fish will be able to detect when robotic fish and marine vessels are approaching them, and these FRS objects may act as stressors, affecting patterns and interactions within the ocean ecosystem.

Thirdly, FRS may alter the environment of fish to such an extent that it steers or alters their natural behaviour [23]. For example, the placement of sensors and their supporting infrastructure can alter the environment’s light and sound levels, leading to light or noise pollution and affecting fish species sensitive to these conditions [23]. Another example of an intrusive sensor infrastructure is the deployment of ‘underwater camera traps’, the remote underwater video (RUV) systems [57]. These are sensor infrastructures specifically designed for monitoring fish. These fish trap cameras are motion-activated; when a fish passes a sensor, they start recording its activity. An even more intrusive sensor infrastructure is the baited remote underwater video (BRUV) [57], which entails installing a camera system on the ocean floor to which bait is attached. As attracted fish move towards the bait, they are recorded. They are promoted as non-invasive methods for monitoring fish and marine wildlife. However, these artefacts essentially invade and shape space in the marine environment, potentially altering and disrupting fish’s natural movement patterns. 

### Relational dimension

FRS mediate the relationship between humans and fish, potentially shaping how we view, relate to, and behave toward them. Therefore, we must consider how FRS will impact the relational dimension of animal-human interactions.

Firstly, one could argue that there has been a power asymmetry between humans and fish since humankind first began catching and eating fish from the seas. Of course, this has been massively exacerbated since the dawn of large industrial fishing vessels, advanced fishing nets, and modern technology, to catch 1.1 to 2.2 trillion individual wild fish a year [53]. Humans also have a dramatic effect on the habitats and livelihoods of fish, decimating their environments, degrading their food sources, and making their habitats unlivable [17, 76]. Therefore, FRS could be seen as the next step in an already tenuous power asymmetry between human beings and the fish they aim to ‘recognise’.

Secondly, the power asymmetry is also evident in the attitudes, framing, and deployment of FRS research. While the literature offers various reasons for deploying FRS for marine conservation, the underpinning reasons often converges in how it views fish and their role in ensuring ecological balance and their ability to maintain the marine ecological environment [38, 82]:

Fish play a critical role in marine ecosystems and make a significant contribution to ecological balance. Effective fish detection can enable researchers to better understand fish population dynamics, migration patterns, and habitat distribution, and to prevent population decline or extinction by formulating effective conservation strategies. [84], p. 1).

The literature often claims that conservation and ecological balance is needed to ensure the sustainable management of fisheries [58], growth and production in the fishing industry [20], ensuring the ‘efficient restoration of marine fisheries resources and ecosystems’ and the ‘scientific management of marine ranching’ [46], p. 1) by ‘preserving fisheries resources and combating overfishing’ [24], p. 1). Subsequent benefits of this would be increasing long-term economic growth [82], providing employment opportunities and ensuring ‘nutritional security of the poor’ [20], p. 603), and helping fish farmers with ‘biomass estimation, disease identification, feeding planning, and cost estimation’ [20].

While some have proposed that deploying FRS should be to preserve biodiversity [3, 5, 7, 8, 58], this is often done with implicit anthropocentric goals. For example, FRS is being deployed to ensure ‘[e]cological surveillance’ [58], maintain the stability of the fish population [82], as a form of underwater management [92], and to ‘help different stakeholders in fisheries, i.e., government officials, policymakers, scientists, and farmers in developing effective conservation measures for these freshwater fish species for stock management’ [20], p. 604). The literature indicates that FRS should be deployed to support human ranching and prevent overfishing, so we can continue benefiting from it in the future [83]. These technologies are implemented to ensure we have sufficient reserves to feed current and future populations, achieve profitable catches for fisheries, and support continued economic growth in the fishing industry (J. [89]). In this context, FRS would be another tool to capture, control, and subjugate the non-human world for human purposes.

Thirdly, and related to this, FRS may inadvertently push the human-fish relationship deeper into a relation of non-human animal objectification. While gathering quantified data for conservation purposes is not new, the employment of AI in this context accelerates and automates the process. It is precisely this automation that may inadvertently risk an increase in the human objectification of fish. The automation of conservation data collection and species recognition would reduce the need for human conservationists to engage directly with the lifeworld of fish, which could have adverse effects on the relationship between humans and fish. When a technology replaces the need for human embodied engagement, the mediating technology becomes the core point of ‘interaction’, and thereby deeply presses its own frame of reference on the relation (Korenhof, [43]).

In the case of FRS, this frame of reference is one of datafication and quantification. FRS can inadvertently foster a human-fish relation in which fish are not encountered as living beings or as things with intrinsic value, but rather are enframed as calculable and determinable entities that can be subjected to calculable management and control [36, 37]. As such, AI-driven FRS may push the human-fish relationship further towards the objectification of fish, framing them as objects to be managed, while information about their lived experience is reduced to data points and measurable parameters, rendering them into ‘quantified animals’ [10]. Moreover, with the corresponding primacy of calculability, the FRS-mediated human-fish relation can easily become a relation of control built upon efficiency and optimisation—the main values that go hand in hand with quantification technologies. A too strong dependency on AI-driven FRS therefore risks producing a human-fish relationship that fails to foster an understanding of and an approach to fish as complex, living beings with their own relations to other living beings and to their natural environment.

The overall results from this section are illustrated in Table 2.

**Table 2:** Concerns arising from each dimension

Dimension	Concerns
Informational	1. There may be biases in the design of FRS, which risk overlooking other important aspects, leading to harm 2. There may be biases in the data used in FRS, which may lead to misallocation of resources, the failure to recognise endangered species, and an inability to take appropriate action when and where needed 3. The use of FRS may be intentionally biased, focusing on certain species while overlooking others. FRS may also be unintentionally biased because of technical or environmental limitations
Infrastructural	1. The deployment of FRS on vessels, robotic fish, drones, and moving cameras may potentially cause physical harm to fish 2. FRS may act as stressors to fish and affect patterns and interactions within the ocean ecosystem 3. FRS, such as the use of underwater camera traps, may alter the environment of fish to such an extent that it steers or alters their natural behaviour
Relational	1. FRS may exacerbate power asymmetries between humans and fish 2. FRS is being deployed with anthropocentric goals in mind (i.e., ensure sustainable catches and ecosystem surveillance) 3. FRS may lead to the quantification of fish, reducing their complexity as living beings with their own relations to other living beings and to their natural environment

## Discussion

This section will focus on responding to the informational, infrastructural, and relational dimensions outlined in the previous section. The following section discusses these three dimensions, offering reflections, suggestions, and recommendations for responding to or overcoming these challenges.

### Informational dimensions discussion

The bias of the information offered by FRS does not necessarily imply that FRS should be abandoned or prohibited. While there are challenges associated with the development and deployment of these technologies in practice, further research and improvements could address and ameliorate these issues. For example, accuracy predictors, identification of potential biases in recommendations, and algorithmic auditing should be implemented into FRS research before it is applied to policy or conservation practice [66]. Independent observations of when the data is collected and/or analysed could improve standards and ensure adherence to best practices.

While these technologies are still very preliminary and have not yet been utilised by policymakers or in conservation decision-making, future developments in FRS should consider the limits and biases of their results and factor in the technical challenges and potential ethical issues that may arise. They may achieve this by raising awareness of their focus on favourable conditions, the fish species, and the need to document these limitations adequately. They should acknowledge species *not* included in their studies that may become endangered or otherwise harmed in the future. Building on the sound scientific basis described above, FRS researchers should provide policymakers with a clear and transparent overview of their research and its limitations, including metadata on what is known and what is unknown. This transparency also refers to translating their results into ways that are communicable and understandable to other disciplines and policymakers.

Thirdly, this transparent communication of the research will enable policymakers and conservationists to take appropriate action to protect endangered fish species and ensure biodiversity. It will allow policymakers to implement appropriate procedures to ensure that FRS is used appropriately for conservation efforts and to develop measures to protect it from misuse or abuse. For example, to ensure that there is no misuse or abuse as a result of FRS, namely, that it is not misused by fisheries, exotic fish poachers, and so forth.

Lastly, it is essential to be aware of potential ‘function creep’ of FRS, i.e., “**an imperceptibly transformative and therewith contestable change in a data-processing system’s proper activity”** [bold original] [42], p. 53). For example, we can imagine that the underwater monitoring infrastructure might be used by actors in the defence industry to support decision-making in underwater warfare or oil companies to plot their next drilling location. Therefore, it is vital to explore how to prevent the misuse of FRS in situations of function creep.

### Infrastructural dimensions discussion

There is a long history of philosophical arguments against interference in animal behaviour, as illustrated by the case of fish-trap cameras. For example, a core idea of Daoism is non-interference in nature, with the Zhuangzi criticising interference in animals [62]. Arne Naess and the deep ecology movement propose that all living things have intrinsic worth and humans should interfere with nature less [56]. Paul Taylor advocated for a biocentric approach, stating that all organisms have intrinsic worth and are teleological centres of life, and interfering with their behaviour violates respect for their good [75]. In addition, some animal ethicists might propose adopting a laissez-faire, hands-off approach to wild animals, letting animals be in nature [21, 63]. Within this approach, there is a strong moral argument against the use of such fish-trap cameras, as they impinge on fish’s freedom. All of these approaches state that it is wrong to interfere with animals in a way that alters their behaviour, which fish trap cameras are guilty of. Further research is needed to identify more effective, less ethically problematic way to photograph fish for FRS.

Second, if cameras are attached to boats already in the water, there may be no *additional* harm caused by FRS, per se. There is also a comparative correlation assumption: just because other similar technologies (e.g., vessels) harm, leak pollutants, cause noise pollution, and collide with fish, this may also occur with FRS, such as drones and robotic fish. However, these technologies may be designed to collect images of fish without similar repercussions; for example, without leachable material, sensors that allow them to avoid collision with fish, and that do not transmit any noise. This issue then appears to be a technical or engineering issue that can, and should, be addressed. Efforts should be made to ensure that any deployed FRS has minimal potential environmental or biological harm in the ocean.

Third, the psychological or emotional impact of FRS on ocean organisms is more difficult to address because there is uncertainty about whether they would cause such harm in practice. Research on stress in fish (Wendelaar Bonga, 1997) has shown that acute noise from boats increases cortisol levels. However, other studies have shown that stress levels between fish living in quiet vs noisy seas were not significantly different, indicating that fish adapt to noisy environments and/or that boat noise poses no threat to survival [59, 70]. However, other studies have shown that particular fish species are more disturbed by boats than others [39]. Perhaps the deployment of *individual* robotic fish, drones, or cameras would have a relatively minimal impact on the emotional lives of most fish species. A camera, drone, or fish robot may not be a significant issue, or, if it is, it will likely be short-lived given the sheer abundance of interactions, predator chases, and food searches that occur daily. However, if the ocean is littered with these foreign objects, the frequency of emotional distress may increase, making it a greater concern. Currently, these technologies are being deployed only minimally, and it is unclear when, or if, they will be deployed on a wide scale. Therefore, concerns about the emotional impact of FRS do not seem like a credible reason for halting their use; however, the rate and expansion of their use need to be carefully considered in the future.

### Relational dimensions discussion

In response to the criticism that FRS is another form of power over fish and perpetuates an anthropocentric rationale for conservation, efforts should be made to reposition and reclaim conservation technologies such as this one to protect species and ecosystems [38]. A greater emphasis needs to be placed on the intrinsic value of species and ecosystems, and an acknowledgement that human beings have a moral obligation to set right some of the wrongs that we have caused to the non-human world. The biases toward protecting fish to manage catches and ensure a sufficient number of species for our aesthetic pleasure or consumption are inherent within research on fishial identification. Therefore, more education about ecocentric, biocentric, and non-anthropocentric viewpoints is needed to reorient the debate toward a conservation ethic grounded in legitimate care for the non-human world.

Secondly, greater interdisciplinary collaboration among FRS technologists and environmental ethicists, sociologists, and conservationists should ensure a more robust repositioning of the case for deploying these technologies. However, this responsibility does not only rest on the shoulders of computer scientists and engineers developing technologies such as FRS. Still, it should come from ethicists, animal welfare and rights advocates, and conservationists. There needs to be engagement with current practices to evaluate their social and ethical implications, done by working alongside and engaging in dialogue with those in the field developing these technologies.

Thirdly, in response to the criticism that FRS is simply another way to encapture and avert our power over nature, viewing it merely as a resource for human consumption, we need to understand technology’s potential while also acknowledging its problematic implications and dangers. Technology is not neutral and will always reveal the world to us in a certain way. It is essential to have an awareness of this revealing and how it changes our perspectives and behaviour. If we understand the non-neutrality of technology and acknowledge its pitfalls, it (e.g., FRS) can offer perspectives and actions that compensate for human shortcomings. For example, employing a ‘designing with non-humans’ approach to FRS could alleviate some objectification and anthropocentricism in its design [54].

Fourthly, many of the issues with FRS are neither new nor necessarily unique to fish. One of the most striking issues is the tendency to view everything as fundamentally measurable, categorisable, and understandable through an amalgamation of data points (i.e., ‘dataism’), a belief that complex biological entities, relationships, dynamics, emotions, and beliefs can be reduced to data. While this is indeed illustrated in and through FRS, the underpinning ideology grounds this application of AI and its deployment as a whole. Issues such as dataism require shifts in ontological perspectives and worldviews. In the context of FRS, this necessitates changes to the categories and views of fish, as well as to how fish are defined and understood. There needs to be a shift in the language used (i.e., away from framing fish as a resource), in the mindset towards fish, and in the rationale for FRS deployment. Perhaps FRS could be used as a source of good by raising the general public’s awareness of fish. FRS could be used in awareness campaigns, in education, and to make fish more present in the animal-human relationship.

The overall discussion points are summarised in Table 3.

**Table 3:** Responses to dimensional challenges

Dimension	Responses
Informational	1. Steps should be taken to reduce harmful biases in FRS 2. FRS research should be transparent about its limitations, including metadata on what is known and what is unknown, and explainable to other disciplines and policymakers 3. Policymakers should implement procedures to ensure FRS is used appropriately for conservation, and it is not misused or abused 4. Action should be taken to ensure that function creep does not occur
Infrastructural	1. Fish trap cameras may infringe upon the freedom of fish and other animals, and should be reconsidered as a way to collect fish images for FRS 2. Precautions should be taken to ensure that FRS has minimal potential environmental or biological harm in the ocean 3. Further research is needed on the potential emotional harm to fish and ocean wildlife caused by the intensive deployment of FRS
Relational	1. There is a need for more discussion on how non-anthropocentric viewpoints can reorient debates in conservation and conservation technologies (such as FRS) toward a legitimate care for the non-human world 2. Interdisciplinary collaboration between FRS technologists and environmental ethicists, sociologists, and conservationists is needed 3. Better accounting of the dangers and risks of FRS should be implemented 4. FRS could be used as a way to raise the general public’s awareness of fish through awareness campaigns, education, and to make fish more present in the animal-human relationship

## Conclusion

Open waters are challenging environments for human agents to gather data. With biodiversity protection high on the sustainable development agenda, FRS can offer relevant insights for corresponding policy development [55]. However, like every technology, FRS is not a neutral tool, but a complex system that works on and intervenes in the world. In this article, we therefore aimed to offer an initial exploration of the implications of FRS in open waters for fish. By focusing specifically on fish, rather than on ethical implications in general (read: implications for humans and their lifeworld), we aimed to draw out the implications of these technical systems for the life they aim to ‘make visible’, a life distant from human life.

In the article, we traced the implications along three dimensions that we believe typify information technologies and help us draw out their implications for the lives with which they intersect: the informational, the infrastructural, and the relational dimensions. Firstly, we discussed how datasets and computer vision algorithms are bound to produce biased information, thereby directing policy decisions in certain directions, potentially favouring some species or regions over others. Secondly, we highlighted how FRS rely on *material* infrastructure that invades and shapes the marine environment, thereby requiring fish to contend with these infrastructures in their natural habitat, potentially disrupting their natural behaviour or even harming them. Thirdly, FRS mediate the relationship between humans and fish. This relational dimension is shaped by both the bias of the informational dimension and the materiality of the infrastructural dimension: FRS produce quantified information about selected fish species or areas, while they are developed, built, maintained, and used by humans. As such, FRS express a *power asymmetry* in the relation between humans and fish that is fundamentally technology-mediated.

In our recommendations, we focused on preventing harm to fish and ensuring species conservation. In the case of bias, this means making the limitations and the focus of the information produced by the FRS transparent and clear, particularly to decision-makers who use these systems for policy development. Along similar lines, the potential environmental benefits of rolling out FRS infrastructure should be proportionate to its impact on fish welfare and the environment. Steps need to be taken to evaluate the potential trade-offs and tensions between FRS’s ecosystemic sustainability and its negative impacts on fish species and their habitats. To counter the possibility that FRS may reduce the human relationship with fish as part of human control over a quantified resource, we recommended acknowledging the system’s risks and supplementing it with more awareness-oriented approaches.

Many of our recommendations are still strongly human-oriented. At best, these systems improve decision-making in biodiversity governance without disrupting or harming fish and their environment. In a perfect world, FRS should contribute to the well-being of fish beyond human goals, means, and decisions. To spark future research, we therefore offer a research agenda that aims to shift FRS’s human-centred approach to a more fish-centred one (see Table 4).

**Table 4:** Shifting from human to fish-centred FRS

FRS Dimension	Human-centred approach	Fish-centred approach
Informational	Human biases cause harm to fish	Greater consideration for all fish species and the ocean ecosystem
Infrastructural	Goal-oriented: serves the goal of gathering and processing fish data	Means-oriented: the infrastructure is designed to improve the living environment for fish
Relational	Fish are a quantified resource for human ends	FRS should be used to understand and build an authentic, caring relationship between humans and fish

FRS is but one of the many AI-based technologies currently emerging in the field of environmental governance and conservation. Overall, these technologies are part of a wider ideology: the belief that, with more data and data-crunching about the planet and life on it, we can better address, mitigate, and adapt to the challenges posed by climate change and biodiversity loss. This article should therefore not be seen as a stand-alone case or research. Nevertheless, by focusing specifically on FRS, this article aims to avoid sweeping claims about AI and to offer marine conservation practitioners food for thought on the appropriateness and proportionality of the systems they design, implement, and use.

## Data Availability

No datasets were generated or analysed during the current study.

## Appendix 1: 30 Articles reviewed

**Table:**

Articles Used in the Literature Review of FSR
Ayyagari, Devi, Corey Morris, Joshua Barnes, and Christopher Whidden. 2023. ‘Toward Low-Cost Automated Monitoring of Life below Water with Deep Learning’. *Environmental Data Science* 2: e13
Bajpai, Abhishek, Naveen Tiwari, Aditya Yadav, Divyansh Chaurasia, and Mohit Kumar. 2024. ‘Enhancing Underwater Object Detection: Leveraging YOLOv8m for Improved Subaquatic Monitoring’. *SN Computer Science* 5 (6): 793
Basuki, Rizqi, and Hustinawaty. 2024. ‘You Only Look Once v8 for Fish Species Identification’. *IAES Int. J. Artif. Intell* 13: 3314–21
Bhanumathi, M., and B. Arthi. 2024a. ‘FishRNFuseNET: Development of Heuristic-Derived Recurrent Neural Network with Feature Fusion Strategy for Fish Species Classification’. *Knowledge and Information Systems* 66 (3): 1997–2038
Bhanumathi, M., and B. Arthi. 2024b. ‘OMSACC-SGRAN: An Implementation of Hybrid Optimized Multi-Scale Atrous Convoluted CNN with Self Guided Residual Attention Network for Fish Species Classification’. *Multimedia Tools and Applications* 83 (39): 87,199–235
Bürgi, Kilian, Charles Bouveyron, Diane Lingrand, Benoit Derijard, Frédéric Precioso, and Cécile Sabourault. 2025. ‘Towards a Fully Automated Underwater Census for Fish Assemblages in the Mediterranean Sea’. *Ecological Informatics* 85 (March): 102,959. https://doi.org/10.1016/j.ecoinf.2024.102959
Chicchon, Miguel, Hector Bedon, Carlos R. Del-Blanco, and Ivan Sipiran. 2023. ‘Semantic Segmentation of Fish and Underwater Environments Using Deep Convolutional Neural Networks and Learned Active Contours’. *IEEE Access* 11: 33,652–65. https://doi.org/10.1109/ACCESS.2023.3262649
Deka, Jayashree, Shakuntala Laskar, and Bikramaditya Baklial. 2023. ‘Automated Freshwater Fish Species Classification Using Deep CNN’. *Journal of The Institution of Engineers (India): Series B* 104 (3): 603–21
Ezzeddini, Lotfi, Jalel Ktari, Tarek Frikha, et al. 2024. ‘Analysis of the Performance of Faster R-CNN and YOLOv8 in Detecting Fishing Vessels and Fishes in Real Time’. *PeerJ Computer Science* 10: e2033
Haider, Adnan, Muhammad Arsalan, Se Hyun Nam, Haseeb Sultan, and Kang Ryoung Park. 2023. ‘Computer-Aided Fish Assessment in an Underwater Marine Environment Using Parallel and Progressive Spatial Information Fusion’. *Journal of King Saud University—Computer and Information Sciences* 35 (3): 211–26. https://doi.org/10.1016/j.jksuci.2023.02.016
Indhumathi, N., S. Maheswaran, I. Bhuvaneshwarri, U. M. Ramya, S. Kayalvili, and S. Aadithyan. 2025. ‘Environmental Management through Machine Learning-Based Fish Species Classification for Sustainable Fisheries’. *Journal of Environmental Nanotechnology* 13 (4): 232–40. https://doi.org/10.13074/jent.2024.12.242651
Jalal, Ahsan, Ahmad Salman, Ajmal Mian, Salman Ghafoor, and Faisal Shafait. 2025. ‘DeepFins: Capturing Dynamics in Underwater Videos for Fish Detection’. *Ecological Informatics* 86 (May): 103,013. https://doi.org/10.1016/j.ecoinf.2025.103013
Li, Shenghong, Peiliang Li, Shuangyan He, et al. 2024. ‘An Automatic Detection and Statistical Method for Underwater Fish Based on Foreground Region Convolution Network (FR-CNN)’. *Journal of Marine Science and Engineering* 12 (8): 1343
Lilkendey, Julian, Cyril Barrelet, Jingjing Zhang, et al. 2024. ‘Herbivorous Fish Feeding Dynamics and Energy Expenditure on a Coral Reef: Insights from Stereo‐video and AI ‐driven 3D Tracking’. *Ecology and Evolution* 14 (3): e11070. https://doi.org/10.1002/ece3.11070
Liu, Mingxin, Wencheng Jiang, Mingxin Hou, Zihua Qi, Ruixin Li, and Chun Zhang. 2023. ‘A Deep Learning Approach for Object Detection of Rockfish in Challenging Underwater Environments’. *Frontiers in Marine Science* 10 (December): 1,242,041. https://doi.org/10.3389/fmars.2023.1242041
Lu, Zhaoxuan, Xiaolong Zhu, Haitao Guo, Xingang Xie, Xiangzi Chen, and Xiangqian Quan. 2024. ‘FishFocusNet: An Improved Method Based on YOLOv8 for Underwater Tropical Fish Identification’. *IET Image Processing* 18 (12): 3634–49. https://doi.org/10.1049/ipr2.13202
Malathi, V., A. Manikandan, and Kalimuthu Krishnan. 2023. ‘Optimzied Resnet Model of Convolutional Neural Network for under Sea Water Object Detection and Classification’. *Multimedia Tools and Applications* 82 (24): 37,551–71
Nabi, M. M., Chiranjibi Shah, Simegnew Yihunie Alaba, et al. 2023. ‘Probabilistic Model-Based Active Learning with Attention Mechanism for Fish Species Recognition’. *OCEANS 2023—MTS/IEEE U.S. Gulf Coast*, September 25, 1–8. https://doi.org/10.23919/OCEANS52994.2023.10337403
Nayak, Ashu, and F. Rahman. 2024. ‘A Deep Learning-Based Intelligent Automatic Detection and Classification of Fish Species in Marine Environment’. *Natural and Engineering Sciences* 9 (3): 144–53
Robillard, Alexander J., Michael G. Trizna, Morgan Ruiz‐Tafur, et al. 2023. ‘Application of a Deep Learning Image Classifier for Identification of Amazonian Fishes’. *Ecology and Evolution* 13 (5): e9987. https://doi.org/10.1002/ece3.9987
Saleh, Alzayat, Marcus Sheaves, Dean Jerry, and Mostafa Rahimi Azghadi. 2024. ‘Applications of Deep Learning in Fish Habitat Monitoring: A Tutorial and Survey’. *Expert Systems with Applications* 238 (March): 121,841. https://doi.org/10.1016/j.eswa.2023.121841
Veiga, Ricardo J. M., and João M. F. Rodrigues. 2024. ‘Fine-Grained Fish Classification From Small to Large Datasets With Vision Transformers’. *IEEE Access* 12: 113,642–60. https://doi.org/10.1109/ACCESS.2024.3443654
Winkler, Julian, Sabah Badri-Hoeher, and Fatna Barkouch. 2023. ‘Activity Segmentation and Fish Tracking From Sonar Videos by Combining Artifacts Filtering and a Kalman Approach’. *IEEE Access* 11: 96,522–29. https://doi.org/10.1109/ACCESS.2023.3294710
Wu, Fei, Yitao Zhang, Lang Wang, Qiu Hu, Shengli Fan, and Weiming Cai. 2023. ‘A Deep Learning-Based Lightweight Model for the Detection of Marine Fishes’. *Journal of Marine Science and Engineering* 11 (11): 2156
Xing, Bowen, Min Sun, Minyang Ding, and Chuang Han. 2023. ‘Fish Sonar Image Recognition Algorithm Based on Improved YOLOv5’. *Mathematical Biosciences and Engineering* 21 (1): 1321–41. https://doi.org/10.3934/mbe.2024057
Yang, Chen, Jian Xiang, Xiaoyong Li, and Yunjie Xie. 2024. ‘FishDet-YOLO: Enhanced Underwater Fish Detection with Richer Gradient Flow and Long-Range Dependency Capture through Mamba-C2f’. *Electronics* 13 (18): 3780
Zhai, Jiping, Lu Han, Ying Xiao, Mai Yan, Yueyue Wang, and Xiaodong Wang. 2023. ‘Few-Shot Fine-Grained Fish Species Classification via Sandwich Attention CovaMNet’. *Frontiers in Marine Science* 10 (March): 1,149,186. https://doi.org/10.3389/fmars.2023.1149186
Zhang, Jiushuang, and Yong Wang. 2024. ‘A New Workflow for Instance Segmentation of Fish with YOLO’. *Journal of Marine Science and Engineering* 12 (6): 1010. https://doi.org/10.3390/jmse12061010
Zhang, Peng, Hong Yu, Haiqing Li, et al. 2023. ‘MSGNet: Multi-Source Guidance Network for Fish Segmentation in Underwater Videos’. *Frontiers in Marine Science* 10 (September): 1,256,594. https://doi.org/10.3389/fmars.2023.1256594
Zouin, Boubker, Jihad Zahir, Florian Baletaud, Laurent Vigliola, and Sébastien Villon. 2024. ‘Improving CNN Fish Detection and Classification with Tracking’. *Applied Sciences* 14 (22): 10,122

## Footnotes

1 These articles were behind paywalls (mostly, IEEE articles, which our university did not have agreements with) and we were unable to retrieve them through online searches or by contacting the authors through ResearchGate.

2 European Commission, *European Digital Twin Ocean*, https://research-and-innovation.ec.europa.eu/funding/funding-opportunities/funding-programmes-and-open-calls/horizon-europe/eu-missions-horizon-europe/restore-our-ocean-and-waters/european-digital-twin-ocean_en. Last accessed 08–07-2026.

3 YOLO is a real-time object detection algorithm that uses a CNN to split an image into specific regions and identify bounding boxes, along with weighted predicted probabilities for each object’s location and class. It was first developed in 2015 and has undergone several revisions, with YOLOv12 being the latest version released in 2025.

4 While there is no clear definition of what a ‘charismatic species’ entails, it points to a species being favoured for conservation purposes over other species by for example being cute, impressive, beautiful, under threat, etc. [22].
